# Tailoring the Implementation of New Biomarkers Based on Their Added Predictive Value in Subgroups of Individuals

**DOI:** 10.1371/journal.pone.0114020

**Published:** 2015-01-26

**Authors:** A. van Giessen, K. G. M. Moons, G. A. de Wit, W. M. M. Verschuren, J. M. A. Boer, H. Koffijberg

**Affiliations:** 1 Julius Center for Health Sciences and Primary Care, University Medical Center Utrecht, The Netherlands; 2 National Institute for Public Health and the Environment, Bilthoven, The Netherlands; Uppsala Clinical Research Center, SWEDEN

## Abstract

**Background:**

The value of new biomarkers or imaging tests, when added to a prediction model, is currently evaluated using reclassification measures, such as the net reclassification improvement (NRI). However, these measures only provide an estimate of improved reclassification at population level. We present a straightforward approach to characterize subgroups of reclassified individuals in order to tailor implementation of a new prediction model to individuals expected to benefit from it.

**Methods:**

In a large Dutch population cohort (n = 21,992) we classified individuals to low (<5%) and high (≥5%) fatal cardiovascular disease risk by the Framingham risk score (FRS) and reclassified them based on the systematic coronary risk evaluation (SCORE). Subsequently, we characterized the reclassified individuals and, in case of heterogeneity, applied cluster analysis to identify and characterize subgroups. These characterizations were used to select individuals expected to benefit from implementation of SCORE.

**Results:**

Reclassification after applying SCORE in all individuals resulted in an NRI of 5.00% (95% CI [-0.53%; 11.50%]) within the events, 0.06% (95% CI [-0.08%; 0.22%]) within the nonevents, and a total NRI of 0.051 (95% CI [-0.004; 0.116]). Among the correctly downward reclassified individuals cluster analysis identified three subgroups. Using the characterizations of the typically correctly reclassified individuals, implementing SCORE only in individuals expected to benefit (n = 2,707,12.3%) improved the NRI to 5.32% (95% CI [-0.13%; 12.06%]) within the events, 0.24% (95% CI [0.10%; 0.36%]) within the nonevents, and a total NRI of 0.055 (95% CI [0.001; 0.123]). Overall, the risk levels for individuals reclassified by tailored implementation of SCORE were more accurate.

**Discussion:**

In our empirical example the presented approach successfully characterized subgroups of reclassified individuals that could be used to improve reclassification and reduce implementation burden. In particular when newly added biomarkers or imaging tests are costly or burdensome such a tailored implementation strategy may save resources and improve (cost-)effectiveness.

## Introduction

Prediction models are increasingly used as an aid in making medical decisions concerning diagnostic, therapeutic and preventive management. In the past three decades many new prediction models have been developed with the aim to improve on existing models. In addition, many existing models have been extended or updated by adding new risk predictors, such as biomarkers or imaging tests, updating predictor weights, or tailoring coefficients to certain populations [1–3].

**Figure 1:**
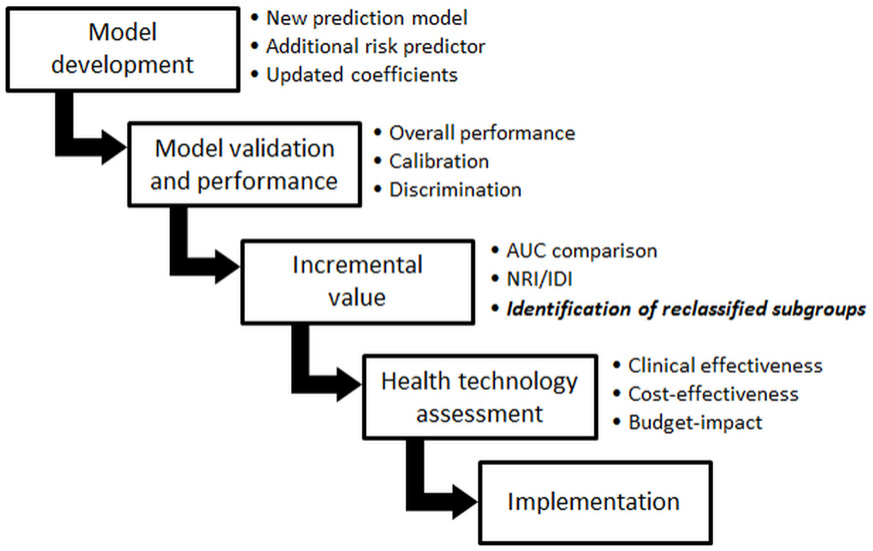
Evaluation process of a new prediction model. Abbreviations: AUC = Area Under the (ROC-) Curve, NRI = Net Reclassification Improvement, IDI = Integrative Discrimination Improvement.

Prior to potential implementation, a new or extended prediction model ought to be evaluated in several stages (Fig. 1) [4–7]. First, its performance is commonly assessed by measures of discrimination and calibration [8]. Subsequently, it is essential to evaluate the incremental value of the new model, as compared to the existing model [9]. Several incremental performance measures are available, such as the difference in the area under the receiver operating characteristic curve, net reclassification improvement (NRI) and integrated discrimination improvement [10]. All these measures give indication of the average improved performance of a new or extended prediction model. However, favourable performance of one prediction model over the other may be the result of improved predictions in one (larger) group of individuals and similar or worse predictions in another group. On top of some individuals receiving worse predictions, performing additional tests in every individual may be undesirable, because of costs and invasiveness of such tests. Hence, there is a clear need to select individuals who actually benefit from a new prediction model, possibly including additional biomarkers or tests.

One way of selecting of individuals is to identify those for whom risk prediction will be improved by application of a new model or addition of tests, for instance through optimization of a window of prediction values [11]. However, more accurate prediction does not result in improved health outcomes if it does not lead to improved patient management. Recent prediction research and literature have clearly adopted this view through the use of the NRI to compare the performance of different prediction models and evaluate the added value of novel risk predictors [8, 9, 12]. Despite its drawbacks the NRI is widely used because of its clinical relevance, as it indicates to what extent a new prediction model improves classification of subjects (with and without the event under study) compared to an existing prediction model, and is therefore likely to also improve treatment decisions, given fixed treatment thresholds [4, 13, 14]. The approach to selection of individuals proposed here follows and expands this focus on improving treatment decisions.

**Figure 2:**
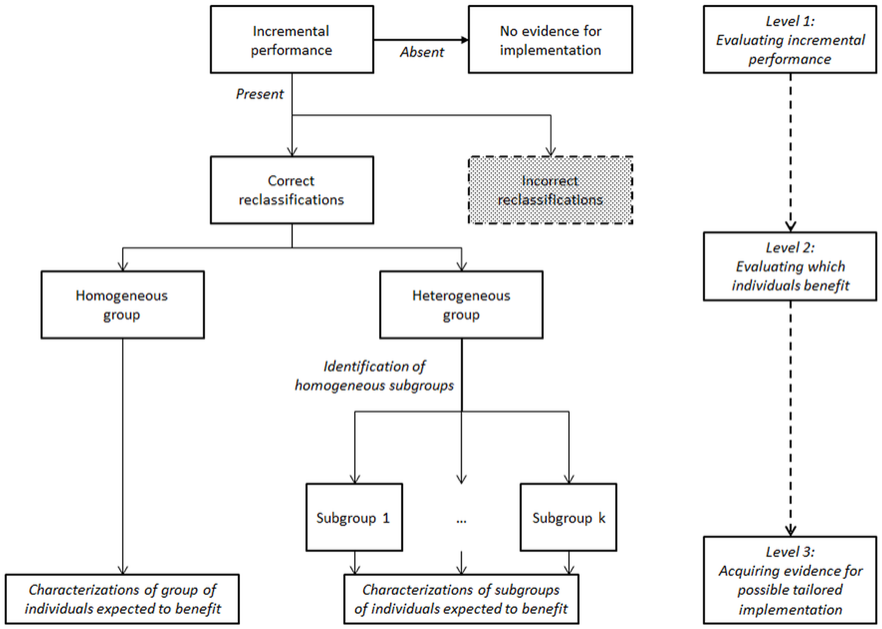
The added value of identifying and characterizing reclassified subgroups. This figure shows that at level 1, assessing the incremental performance, a new prediction model or risk predictor may be selected for implementation in the general population. At level 2, the correctly reclassified individuals are inspected. The additional step, level 3, of identification and characterization of typically reclassified subgroups allows for more informed decision and provides evidence for possible tailored implementation. Actual implementation will then depend on the effectiveness and cost-effectiveness per subgroup.

We propose an additional step when evaluating a new prediction model or risk predictor: to further characterize (subgroups of) reclassified individuals using cluster analysis (Fig. 1). Having additional information on what types of individuals are correctly reclassified indicates who might benefit when introducing a new prediction model or risk predictor. Such knowledge of reclassification impact on subgroup level allows tailored implementation of new prediction models, biomarkers or imaging tests, by applying them only in subgroups of individuals expected to benefit from it, which may improve (cost-)effectiveness of risk-based strategies (Fig. 2).

Subgroups expected to benefit can be identified for any possible adaptation of a prediction model, such as additional biomarkers or imaging tests, but also the introduction of a new prediction model. In this study, we use an empirical example of the latter situation, comparing cardiovascular disease (CVD) risk prediction by two competing risk scores; the Framingham risk score (FRS) and the Systematic Coronary Risk Evaluation (SCORE), to demonstrate the feasibility of our approach [15]. The FRS for CVD risk prediction was developed in 1991 [15]. Since then, model updates have been done and completely new models have been developed, such as the Prospective Cardiovascular Münster (PROCAM) score, the QRESEARCH cardiovascular risk (QRISK) algorithms, and SCORE [2, 3], [16–18]. The incremental performance of SCORE compared to the FRS has been assessed in previous studies, mostly in favor of SCORE [19], but it is unclear which individuals have benefit from these new prediction models. Using data from a large prospective cohort study we evaluate the incremental performance of SCORE as compared to the FRS and illustrate how to characterize the individuals that are correctly and incorrectly reclassified when replacing the FRS with SCORE [20]. Furthermore, we demonstrate how these subgroup characterizations may be used for tailored implementation of a new prediction model only in subgroups of individuals expected to benefit.

## Methods

### Identification and characterization of reclassified subgroups

Using individual participant data (IPD), predicted risks per individual according to an existing and new prediction model can be assessed as well as their performance and incremental performance [8]. Predefined risk categories, preferably recommended by guidelines [12], then allow for estimation of the number of individuals being reclassified, correctly or incorrectly, and reclassification measures. Evaluating which individuals are expected to benefit from additional biomarkers or a new prediction model, we aim to characterize the correctly reclassified individuals (Fig. 2). When evaluating an additional (non-correlated) predictor this may be a rather homogenous group, which allows it to be characterized by a single (set of) characteristic(s). In case of the addition of multiple or correlated biomarkers, or comparison with a new prediction model, the group of correctly reclassified individuals may be very heterogeneous. In this case, for sufficiently large groups of reclassified individuals more homogenous subgroups can be identified, for example by application of cluster analysis, allowing characterization.

In brief, cluster analysis methods explore data to discover clusters, i.e. subgroups, of individuals who are similar to each other and different from individuals in other clusters as defined by a similarity measure [21–23]. No general best approach to cluster analysis exists and choices regarding the clustering method should be made dependent on features of the data [21]. Given that different choices in e.g. similarity measure or number of clusters may lead to differences in the (number of) detected clusters, cluster validation is essential in finding the clustering that best fits the underlying data [24, 25]. Cluster validation can be performed by assessing the quality of the cluster solution, i.e., the identified set of clusters, and by comparison with cluster solutions obtained when replicating the analysis on different (sub)samples of the data to assess the robustness of the solution [21].

Based on the characterizations of the correctly reclassified subgroups, similar individuals, expected to benefit from a new prediction model (or additional biomarker or imaging test), can be selected. Many methods of selecting these individuals exist, where one simple way is just by evaluating if their characteristics appear to match the ranges of characteristics observed in the correctly reclassified subgroups. The implementation of the new prediction model can then be tailored by applying it only to these selected individuals who are expected to benefit.

### Characterization of reclassified subgroups when replacing the FRS with SCORE

Our empirical illustration, which aims to characterize the individuals reclassified if their FRS-based risk estimate is replaced by a SCORE-based risk estimate, uses data from ‘The Monitoring Project on Chronic Disease Risk Factors’ (MORGEN). This large-scale monitoring project was carried out between 1993 and 1997 among men and women living in Amsterdam, Doetinchem and Maastricht, The Netherlands [20]. From this cohort (n = 21,992) we excluded individuals with prevalent cardiovascular disease (n = 264) and those who have not given informed consent (n = 1,435) for linkage to registry data (not mutually exclusive). As our aim was only to provide an illustration of the approach, and not so much to choose between the two risk models for the Dutch situation, individuals with any missing value (n = 658) were also excluded.

The FRS for fatal CVD risk includes the predictors gender, age, systolic blood pressure (SBP), total cholesterol (TC), HDL-cholesterol (HDL-C), presence of left ventricular hypertrophy on an electrocardiogram (ECG-LVH), and smoking and diabetes status [15]. Information on ECG-LVH was not available and was set to ‘absent’ given the low prevalence (2.9% of men and 1.5% of women in the Framingham Heart Study) [26]. As incremental performance, and especially reclassification measures, are dependent on the calibration of the model in the IPD [27], we calibrated the FRS to the MORGEN cohort (see S1 Text. Definition of fatal cardiovascular events for definition of fatal CVD, see S2 Text. Prediction model performance for discrimination and calibration details) [15, 28]. The calibrated FRS was used to calculate the predicted 10-year fatal CVD risk for each individual in the MORGEN cohort (9,168 men and 10,947 women. Finally, individuals were classified into the low (<5%) and high (≥5%) risk category [29].

SCORE exists of two different models; SCORE-high and SCORE-low, both using the predictors gender, age, SBP, TC, and smoking status [18]. Here we used the SCORE-low model, because it had the best calibration on the Dutch population, similar to the MORGEN cohort, in previous studies [30]. Recalibration was not necessary (see S2 Text. Prediction model performance for discrimination and calibration details). The fatal CVD risk for each individual was then calculated using SCORE-low and individuals were again classified into the low and high-risk category. Subsequently, the numbers of correctly and incorrectly upward and downward reclassified individuals across the defined risk categories were assessed and the survival (or prospective) NRI, using Kaplan-Meier estimates for censored observations, was calculated [18, 29, 31].

Cluster analysis was then applied to the reclassified groups of substantial size to identify which subgroups of individuals were typically reclassified. We applied the TwoStep Cluster method, available in SPSS, using a likelihood similarity measure, which can handle a mix of continuous and discrete variables, present in both prediction models [32–34]. To improve cluster solution stability we included at most *k* variables if a group of reclassified individuals contained at least *2^k^* individuals [35]. We selected these *k* variables from the predictors present in both the FRS and SCORE and used backward selection to eliminate those variables with the lowest importance for clustering. We used the Bayesian Information Criterion (BIC) to select the number of clusters and applied an outlier detection setting of 25%.

The quality of the cluster solutions was assessed using the average silhouette width, where a good (0.5;1], fair (0.25;0.5] or poor [-1;0.25] value indicates that strong, weak or no substantial structure, respectively, has been found [23, 32]. Robustness was assessed by replicating the analysis using 1,000 bootstrap datasets [36–38]. To compare the original subgroups to subgroups identified in bootstrap samples, the adjusted Rand index was calculated for each sample using R and the *mclust* package [21, 39–41]. For randomly chosen subgroups this index would have value 0, whereas for perfectly identical subgroups its value would be 1.

### Tailored implementation of SCORE in subgroups expected to benefit

Based on the characterizations of the subgroups of correctly reclassified individuals matching individuals were selected. Individuals are selected if they fulfill the multivariate criterion of having equal binary risk factor values (gender, smoking, and diabetes) and having continuous risk factor values (age, SBP, TC and HDL-C) all falling within the range of 2 standard deviations of the corresponding mean values for one of the correctly reclassified subgroups. If a subgroup cannot be characterized by a binary risk factor, for instance because it consists of 75% smokers, this characteristic was not incorporated into the selection criterion.

Implementation of SCORE-low was then tailored to those individuals who are expected to benefit by applying it only to the selected individuals, i.e. those who could be classified to one of the defined subgroups. Subsequently, the numbers of correctly and incorrectly upward and downward reclassified individuals across the defined risk categories were reassessed and the (survival type) NRI was recalculated. We used bootstrapping (n = 1,000) to repeat the selection of individuals and to estimate confidence intervals of the NRI of the tailored implementation.

## Results

### Initial reclassification results

The recalibrated FRS classified 19,745 individuals into the low (<5%) and 370 individuals into the high-risk category (≥5%). SCORE-low classified 19,755 individuals into the low and 360 individuals into the high-risk category. Replacing the recalibrated FRS with SCORE-low, 234 individuals were reclassified of which 8 upward and 2 downward within the events and 104 upward and 120 downward within the non-events (table 1). This resulted in an NRI of 5.00% (95% CI [-0.53%;11.50%]) within the events, 0.06% (95% CI [-0.08%;0.22%]) within the nonevents, and a total NRI of 0.051 (95% CI [-0.004;0.116]).

**Table 1:** Reclassification with SCORE-low instead of FRS in all individuals.

***Without events***	***SCORE-low***		***Total without events***	***Number (%)***
***Recalibrated FRS***	**Low-risk (<5%)**	**High-risk (≥5%)**	No change	19,771 (98.88%)
**Low-risk (<5%)**	19,537	104	Up classification	104 (0.52%)
**High-risk (≥5%)**	120	234	Down classification	120 (0.60%)
***With events***	**SCORE-low**		*Total with events*	*Number (%)*
***Recalibrated FRS***	**Low-risk (<5%)**	**High-risk (≥5%)**	No change	110 (91.67%)
**Low-risk (<5%)**	96	8	Up classification	8 (6.67%)
**High-risk (≥5%)**	2	14	Down classification	2 (1.67%)
***Observed KM estimates***	**SCORE-low**		All individuals	Number (%)
***Recalibrated FRS***	**Low-risk (<5%)**	**High-risk (≥5%)**	No change	19,881 (98.84%)
**Low-risk (<5%)**	0.29%	4.58%	Up classification	112 (0.56%)
**High-risk (≥5%)**	1.27%	3.76%	Down classification	122 (0.61%)

Abbreviations: FRS = Framingham Risk Score, KM = Kaplan Meier

This table shows the distribution of the 20,115 individuals with and without events in the MORGEN-cohort across risk categories. Individuals with and without events were all classified according to their 10-year absolute risk to develop a fatal cardiovascular disease event with the Framingham Risk Score or all classified with SCORE-low. The bottom rows show the observed 10-year Kaplan-Meier absolute risk estimates for all individuals (with and without events).

### Characterization and cluster analysis of reclassified individuals

Overall, the groups of reclassified individuals contained large variation in risk factor levels (table 2). Given their small size, the incorrectly downward reclassified group (n = 2, table 2D) was not further subdivided and the correctly upward reclassified group (n = 8, table 2A) was only subdivided in men and women. Cluster analysis was performed on the 104 incorrectly upward and 120 correctly downward reclassified individuals (table 2B, C). Both groups contained sufficient individuals to include six risk factors in the cluster analysis. The five predictors present in both the FRS and SCORE-low were used as well as diabetes status, selected by its cluster predictor importance. In both reclassified groups the BIC selected 3 clusters, outliers were not detected.

**Table 2:** Characterizations of reclassified individuals from the MORGEN cohort.

*A.* *Subgroups of correctly upward reclassified individuals (n = 8) ^a^*									
**Subgroup**	**# individuals**	**Male**	**Age (yrs)**	**TC (mmol/L)**	**SBP (mmhg)**	**Smoking**	**Diabetes**	**HDL-C (mmol/L)**	**Predicted FRS risk (%)**
			**mean (SD)**	**mean (SD)**	**mean (SD)**			**mean (SD)**	**mean (SD)**
*Total*	*8 (100%)*	*50.0%*	*61.3 (4.1)*	*6.1 (1.2)*	*150.0 (19.9)*	*75.0%*	*0.0%*	*1.3 (0.2)*	*4.4 (0.7)*
A1	4 (50.0%)	100.0%	58.7 (4.3)	6.2 (1.7)	141.8 (16.6)	75.0%	0.0%	1.2 (0.1)	4.8 (0.1)
A2	4 (50.0)	0.0%	64.0 (1.4)	6.1 (0.8)	158.3 (21.6)	75.0%	0.0%	1.4 (0.3)	4.1 (0.9)
*B.* *Subgroups of incorrectly upward reclassified individuals (n = 104)*										
**Subgroup**	**# individuals**	**Male**	**Age (yrs)**	**TC (mmol/L)**	**SBP (mmhg)**	**Smoking**	**Diabetes**	**HDL-C^b^ (mmol/L)**	**Predicted FRS risk (%)**
			**mean (SD)**	**mean (SD)**	**mean (SD)**			**mean (SD)**	**mean (SD)**
*Total*	*104 (100%)*	*85.6%*	*59.4 (3.7)*	*6.1 (1.1)*	*152.7 (24.5)*	*65.4%*	*9.6%*	*1.6 (0.4)*	*4.0 (0.8)*
B1	29 (27.9%)	100.0%	60.6 (2.9)	5.8 (1.1)	166.9 (17.5)	0.0%	0.0%	1.6 (0.4)	4.0 (0.8)
B2	15 (14.4%)	0.0%	62.2 (2.8)	7.0 (1.2)	169.2 (29.3)	53.3%	0.0%	1.6 (0.4)	3.7 (0.6)
B3	60 (57.7%)	100.0%	58.1 (3.8)	6.0 (1.0)	141.7 (20.1)	100.0%	12.9%	1.5 (0.5)	4.0 (0.8)
*C.* *Subgroups of correctly downward reclassified individuals (n = 120)*										
**Subgroup**	**# individuals**	**Male**	**Age (yrs)**	**TC (mmol/L)**	**SBP (mmhg)**	**Smoking**	**Diabetes**	**HDL-C^b^ (mmol/L)**	**Predicted FRS risk (%)**
			**mean (SD)**	**mean (SD)**	**mean (SD)**			**mean (SD)**	**mean (SD)**
*Total*	*120 (100%)*	*84.4%*	*58.1 (3.8)*	*6.2 (1.2)*	*145.4 (16.0)*	*44.2%*	*20.8%*	*0.9 (0.2)*	*6.3 (1.7)*
C1	50 (41.7%)	100.0%	60.0 (3.3)	6.1 (1.0)	148.0 (13.5)	0.0%	0.0%	0.9 (0.2)	6.0 (1.0)
C2	29 (24.2%)	34.5%	58.5 (3.0)	6.2 (1.3)	153.8 (16.8)	41.4%	86.2%	0.9 (0.2)	7.2 (2.3)
C3	41 (34.2%)	100.0%	55.5 (3.4)	6.2 (1.3)	136.4 (14.0)	100.0%	0.0%	0.8 (0.1)	5.9 (1.8)
*D.* *Subgroups of incorrectly downward reclassified individuals (n = 2) ^c^*										
**Subgroup**	**# individuals**	**Male**	**Age (yrs)**	**TC (mmol/L)**	**SBP (mmhg)**	**Smoking**	**Diabetes**	**HDL-C (mmol/L)**	**Predicted FRS risk (%)**
*Total*	*2 (100%)*	*[1;1]*	*[58.4;57.9]*	*[5.3;6.9]*	*[162;157]*	*0.0%*	*0.0%*	*[0.8;1.0]*	*[6.2;5.5]*

*Abbreviations:* TC = total cholesterol, HDL-C = HDL-cholesterol, SBP = systolic blood pressure.

Characteristics for the total reclassified groups and each subgroup are given as the mean (standard deviation) for continuous risk factors and as percentage for the dichotomous variables. For consistency, this representations was used for every group. However, for small groups these values may be uncertain.

^a.^The group of correctly upward reclassified individuals was, because of its small size, only subdivided in men and women.

^b.^HDL-C was not used in the cluster analysis.

^c.^Since the group of incorrectly downward reclassified individuals only included two individuals it was not further subdivided and parameter values were given for both individuals instead of means and standard deviations.

The clusters are clearly distinct in certain risk factors, whereas in others differences may be less apparent (table 2). Among the incorrectly upward reclassified individuals subgroup B1 (27.9%) consisted of nonsmoking, non-diabetic men with high mean SBP (table 2B). Subgroup B2 made up 14.4% and contained non-diabetic, women with high mean SBP and TC. Finally, the large subgroup B3 (57.7%) consisted of smoking men, who were on average younger than the other subgroups and had much lower SBP. Among the correctly downward reclassified individuals a large subgroup, C1 (41.7%), consisted of non-smoking, non-diabetic men (table 2C). Subgroup C2 made up 24.2% and contained individuals almost all having diabetes and among whom smoking was common (41.4%). Finally, subgroup C3 (34.2%) consisted of smoking men, who were on average younger than the other subgroups and had much lower SBP. A full characterization and validation results of the subgroups can be found in table 2 and S3 Text. Characterization and validation of identified subgroups.

### Tailored implementation to subgroups

Based on the characteristics of the correctly reclassified subgroups (table 2), 2,707 (12.3%) individuals in our cohort fulfilled the multivariate criterion of having equal binary risk factors and all continuous risk factor values within the range of 2 standard deviations of the means of (at least) one of the correctly reclassified subgroups. The selection consisted of 764 individuals complying with the ranges of subgroup A1, and 196, 482, 1,068, and 197 complying with the ranges of subgroups A2, C1, C2, and C3, respectively (not mutually exclusive). Hence, these individuals were selected for tailored implementation. They contained 90.8% of the originally correctly downward and all correctly upward reclassified individuals as well as all incorrectly downward and 57.7% of the incorrectly upward reclassified individuals when SCORE-low was applied to every individual in the MORGEN cohort.

### Reclassification results after tailored implementation

Applying SCORE-low instead of the recalibrated FRS only in the 2,707 selected individuals (12.3%), 170 individuals were reclassified of which 8 upward and 2 downward within the events and 60 upward and 109 downward within the non-events (table 3). This resulted in an NRI of 5.32% (95% CI [-0.13%;12.06%]) within the events, 0.24% (95% CI [0.10%;0.36%]) within the nonevents, and a total NRI of 0.055 (95% CI [0.001;0.123]). Overall, the risk levels for individuals reclassified by tailored implementation of SCORE were more accurate (table 3).

**Table 3:** Reclassification with SCORE-low instead of FRS in subgroups expected to benefit.

***Without events***	**SCORE-low**		***Total without events***	***Number (%)***
***Recalibrated FRS***	**Low-risk (<5%)**	**High-risk (≥5%)**	No change	19,826 (99.16%)
**Low-risk (<5%)**	19,581	60	Up classification	60 (0.30%)
**High-risk (≥5%)**	109	245	Down classification	109 (0.55%)
***With events***	**SCORE-low**		*Total with events*	*Number (%)*
***Recalibrated FRS***	**Low-risk (<5%)**	**High-risk (≥5%)**	No change	110 (91.67%)
**Low-risk (<5%)**	96	8	Up classification	8 (6.67%)
**High-risk (≥5%)**	2	14	Down classification	2 (1.67%)
***Observed KM estimates***	**SCORE-low**		All individuals	Number (%)
***Recalibrated FRS***	**Low-risk (<5%)**	**High-risk (≥5%)**	No change	19,936 (99.11%)
**Low-risk (<5%)**	0.29%	7.90%	Up classification	68 (0.34%)
**High-risk (≥5%)**	1.41%	3.59%	Down classification	111 (0.55%)

Abbreviations: FRS = Framingham Risk Score, KM = Kaplan Meier

This table shows the distribution of the 20,115 individuals with and without events in the MORGEN-cohort across risk categories. Individuals with and without events were classified according to their 10-year absolute risk to develop a fatal cardiovascular disease event with the Framingham Risk Score and selected subgroups expected to benefit were reclassified by SCORE-low. The bottom rows show the observed 10-year Kaplan-Meier absolute risk estimates for all individuals (with and without events).

## Discussion

This study shows that application of cluster analysis is a feasible approach to characterize subgroups of reclassified individuals, taking evaluation of prediction models beyond reclassification tables and measures. Furthermore, the characterized subgroups can be used as the starting point for evidence-based tailored implementation of new prediction models, biomarkers and tests, as demonstrated in our empirical example. This approach can be applied to any reclassification result, using nested or non-nested models, with the correctly reclassified subgroups providing information useful to select individuals expected to benefit from the new model, and the incorrectly reclassified subgroups providing information useful to exclude individuals from the new model.

Here, we focused on individuals expected to benefit from the new model only, as in practice new biomarkers and tests quite often are costly or potentially burdensome for individuals (Fig. 2). In such a context, limiting their application to individuals that may be expected to actually benefit from them will save costs and may reduce health loss by reducing unnecessary use. In addition, the characterization of reclassified subgroups may also encourage tailored prediction model development and impact studies of prediction models on health outcomes (and cost-effectiveness of care) may provide more accurate results when accounting for the identified characteristics of reclassified subgroups instead of assuming that all reclassified individuals are similar [6, 42, 43].

When tailoring the implementation of a new prediction model to selected subgroups of individuals expected to benefit, the proportion of these individuals that will indeed be correctly reclassified depends on two factors. First, for two individuals with a similar risk profile it is possible that the new model reclassifies one of these individuals correctly and one incorrectly. Consequently, there may be individuals that match the characterization of a correctly reclassified subgroup, but are themselves incorrectly reclassified. However, our selection criterion is based only on the subgroup characterizations of correctly reclassified individuals. Therefore, applying the new model in individuals matching these characterizations is likely to yield a (much) better balance of correctly reclassified and incorrectly reclassified individuals than applying the new model in everyone. Second, this proportion will depend on the strictness of the selection criterion. In our illustration, we chose a range of 2 standard deviations. Narrowing this to, for instance, 1 standard deviation will select fewer individuals and is likely to further improve the balance between correctly and incorrectly reclassified individuals within this group. Choosing this strictness will be a trade-off between improved reclassification and the costs of the new biomarker.

The results of the cluster analyses may be used in many ways to select individuals for inclusion or exclusion of the new test or biomarker. Here, we chose to simply base our selection on the ranges of risk factor values in the relevant subgroups. Further optimization of the selection process might still be possible and could result in a larger NRI. Finally, we assessed the performance of our tailored approach using the NRI, while more informative alternatives may be available [13]. However, for other measures of compared performance we expect tailoring would still result in improved health effects at reduced costs.

### The empirical example

In our illustration, where individuals from the large Dutch MORGEN cohort were classified by the (recalibrated) FRS and reclassified by SCORE-low, the reclassification table (including the observed risks, table 3) and NRI of 0.051 may have suggested replacement of the FRS for, in this case, the entire Dutch population. Inspection of the reclassification tables showed that reclassifications mainly consist of a group of individuals being correctly reclassified downwards and incorrectly reclassified upwards. When additionally evaluating which individuals benefit from the application of SCORE-low, identifying subgroups among the correctly reclassified individuals, using cluster analysis, could further characterize these individuals.

Furthermore, it was demonstrated that the characterizations of the correctly reclassified subgroups of individuals can be used to select individuals who are expected to benefit from a new prediction model. Replacing the (recalibrated) FRS with SCORE-low in only those individuals expected to benefit slightly increased the NRI within the events and within the non-events, while at the same time reducing the implementation burden by 87.7%. In this particular example the benefits of tailored implementation may be limited, as the application of SCORE-low instead of the FRS does not result in a large implementation burden through costly or invasive measurement of additional risk factors. In general, however, tailoring the implementation of extended prediction models including an expensive or invasive test to those individuals expected to be correctly reclassified with such a test would improve the cost-effectiveness of these prediction models compared to addition of the test for everyone, which may not even be (cost-)effective as a result of the large number of non-beneficial tests performed [44–47].

Although the empirical example shows two risk categories, the presented tailoring approach is easily extendable to multiple risk categories. Commonly, there is agreement about what treatment (if any) should be provided to individuals at low or high risk, while there is uncertainty about the treatment strategy for those at intermediate risk. Therefore, additional tests are often provided to intermediate-risk individuals only, as these might benefit by being reclassified to the low or high-risk category, offering them valid and appropriate treatment. Nevertheless, the intermediate risk category often comprises a large and heterogeneous group of individuals, not all of whom will actually benefit from additional tests and some of whom may even be reclassified incorrectly, resulting in a substantial number of examples of this strategy not being (cost-)effective [44–47]. Altogether, our method extends risk-based tailoring by selecting individuals based on their risk profiles instead of their predicted risk, and by linking the impact of tailored implementation to actual expected improvements in treatment decisions with corresponding improvements in health outcomes and reductions in costs.

### The relevance and application of cluster analysis

In our illustration changes in predicted risk for groups of individuals with diabetes or extreme values of HDL-C may have been expected as these predictors are included in the FRS but not in SCORE-low. This will be similar for adding any other (strong) risk factor to a prediction model. However, changes in predicted risk from both models cannot easily be estimated as these depend on the occurrence of predictors, their coefficients and their correlation. This rules out a-priori identification of reclassified individuals and means that only a post-hoc evaluation, after reclassification, will be able to provide information on relevant subgroups expected to be reclassified. Cluster analysis provides one way of structured identification of such subgroups, but alternative methods are available as well [48–50]. For this illustration we have chosen to include in the cluster analysis only those predictors included in the two prediction models, as these are certainly available when comparing two or more prediction models. If data on other characteristics are available, however, that could contribute to subgroup identification, these could be included in the cluster analysis as well.

### Limitations

Performing cluster analyses additional to the estimation of reclassification measures when comparing models, the use of many cluster variables requires relatively large groups of reclassified individuals and therefore sufficiently large IPD datasets including evidence on all predictors and outcomes [35]. Furthermore, identified subgroups and their prevalence are representative of the data they were derived from. As we propose implementing this approach as an *additional* step in the development and evaluation process of prediction models (Fig. 1), appropriate IPD will generally be available. In situations in which the groups of reclassified individuals are small, subgroup analyses may not be worthwhile, but description of the overall characteristics of these reclassified groups, such as shown in table 2 (top rows), will still be valuable.

As applies to many statistical approaches, also the results of cluster analyses depend on various decisions, such as the choice of the clustering method and the number of subgroups. Accordingly, different subgroups may be identified in the same data by different researchers. This issue can be mitigated, however, through cluster validation, ensuring a stable cluster solution that best fits the data [25]. Cluster analysis may also classify some individuals as outliers, not part of any subgroup. In practice this will not be a problem as only the risk profiles of relatively large reclassified subgroups are of interest and may influence the implementation decision. Similar to prediction modeling, subgroup characterizations should ultimately be externally validated to test their generalizability and assess whether tailored implementation is beneficial in other populations.

We have validated the entire procedure, using appropriate methods, in two steps. First, we validated the cluster solution on its quality using the average silhouette width and on its robustness through bootstrapping and calculating the Rand index, because tailored implementation can only be considered for subgroups based on valid clusters. If, for instance, unstable clusters are identified further investigation of the impact of tailored care based on such clusters is not useful. Second, following cluster validation, individuals were allocated to the corresponding subgroups, and individuals were selected in which the new prediction model is implemented. We used bootstrapping to repeat this second step and to estimate confidence intervals of the NRI of the tailored implementation. Another approach would be to validate the entire procedure at once, capturing all potential sources of variation in all steps. However, this would mean that a divergent number of clusters, as well as non-valid subgroups, may be incorporated.

## Conclusion

When comparing two or more prediction models, or estimating the added value of new predictors (e.g. biomarkers or imaging test results), we recommend to characterize the groups of reclassified individuals. For sufficiently large, heterogeneous reclassified groups, a straightforward application of cluster analysis can identify and characterize subgroups. Such subgroup characterization provides additional insight into the impact of implementing a certain prediction model, beyond existing reclassification summary measures and reclassification tables, and allows tailored implementation in specific subgroups of individuals.

## Supporting Information

S1 Text Definition of fatal cardiovascular events. (DOCX)

S2 Text Prediction model performance. (DOCX)

S3 Text Characterization and validation of identified subgroups. (DOCX)
