# Supplementary material for: Tailoring the Implementation of New Biomarkers Based on Their Added Predictive Value in Subgroups of Individuals
Source: PLoS One. 2015 Jan 26;10(1):e0114020. doi: 10.1371/journal.pone.0114020 (PMC4306488; doi:10.1371/journal.pone.0114020)
Supplement: S1 Text — (DOCX) [file pone.0114020.s001.docx]

**Text S1. Definition of fatal cardiovascular events**

Causes of death in the MORGEN cohort have been coded according to the Ninth Revision of the International Classification of Diseases (ICD-9) until 1996, and after that according to the Tenth Revision of the International Statistical Classification of Diseases (ICD-10). Morbidity data were coded according to ICD-9. Cardiovascular disease events comprised those events with ICD-9: 410-414, 427.5, 428, 415.1, 443.9, 430-438, 440-442, 444, 798.1,798.2, 798.9 or ICD-10: I20-I26, I46, R96, G45, I60-I67, I69, I70-I74, I50.
