# Supplementary material for: Tailoring the Implementation of New Biomarkers Based on Their Added Predictive Value in Subgroups of Individuals
Source: PLoS One. 2015 Jan 26;10(1):e0114020. doi: 10.1371/journal.pone.0114020 (PMC4306488; doi:10.1371/journal.pone.0114020)
Supplement: S2 Text — (DOCX) [file pone.0114020.s002.docx]

**Text S2. Prediction model performance**

The Framingham risk score (FRS) was recalibrated to the observed 10-year fatal CVD risk in the MORGEN cohort, separately for men and women [50,15]. The original FRS applied to the MORGEN data had a calibration slope of 0.56, a baseline risk of 110 per 100,000 (27 per 100,000 for women), and the Hosmer-Lemeshow test gave a p-value <0.001. After recalibration, the calibration slope was 0.80, the baseline risk was 116 per 100,000 (63 per 100,000 for women), and the Hosmer-Lemeshow test gave a p-value of 0.93. The calibration of SCORE-low to the MORGEN data was characterized by a calibration slope of 0.73 and the Hosmer-Lemeshow test gave a p-value of 0.72. Further results of overall performance, discrimination, and calibration can be found in the table below.

| Performance measure | FRS | Calibrated FRS | SCORE-low |
| --- | --- | --- | --- |
| Overall |  |  |  |
| Brier score | 0.006 | 0.006 | 0.006 |
| Discrimination |  |  |  |
| AUC [95% CI] | 0.84 [0.80;0.87] | 0.84 [0.80;0.87] | 0.83 [0.79;0.86] |
| Calibration |  |  |  |
| Calibration slope | 0.56 | 0.80 | 0.73 |
| H-L test | Chi-square 50.1  p<0.001 | Chi-square 3.1  p=0.93 | Chi-square 5.4  p=0.72 |

*Abbreviations: FRS = Framingham risk score, AUC = Area under the ROC-curve, CI = confidence interval, H-L test = Hosmer Lemeshow test with 10 groups.*
