# Supplementary material for: Tailoring the Implementation of New Biomarkers Based on Their Added Predictive Value in Subgroups of Individuals
Source: PLoS One. 2015 Jan 26;10(1):e0114020. doi: 10.1371/journal.pone.0114020 (PMC4306488; doi:10.1371/journal.pone.0114020)
Supplement: S3 Text — (DOCX) [file pone.0114020.s003.docx]

**Text S3. Characterization and validation of identified subgroups**

Among the incorrectly upward reclassified individuals subgroup B1 (27.9%) consisted of nonsmoking, non-diabetic men with a mean age of 60.6 (SD 2.8) and high mean SBP of 166.0 (SD 17.5) (table 2B). Subgroup B2 made up 14.4% and contained non-diabetic, older women with a mean age of 62.2 (SD 2.8), high mean SBP of 169.2 (SD 29.3) and high mean TC of 7.0 (SD 1.2). Finally, the large subgroup B3 (57.7%) consisted of smoking men of which 12.9% had diabetes.

The quality of the cluster solutions was assessed using the average silhouette width, where a good (0.5;1], fair (0.25;0.5] or poor [-1;0.25] value indicates that strong, weak or no substantial structure, respectively, has been found [23,31]. This cluster solution had an average silhouette score of 0.6 corresponding to a ‘good’ cluster solution. Robustness was assessed by replicating the analysis using 1,000 bootstrap datasets [35-37]. The adjusted Rand index was calculated [21,38-40]. For randomly chosen subgroups this index would have value 0, whereas for perfectly identical subgroups its value would be 1. In the bootstrap samples the BIC selected 2, 3, or 4 clusters as the optimum number in 22.8%, 71.9%, and 5.3% of the samples, respectively. In 92.4% of the bootstrap samples no outliers were detected and <1% outliers were detected in the remaining 7.6% of the samples. The adjusted Rand index averaged over all bootstrap samples was 0.94 (SD 0.09), implying that subgroups are highly identical.

Among the correctly downward reclassified individuals a large subgroup, C1 (41.7%), consisted of non-smoking, non-diabetic men with a mean age of 60.0 (SD 3.3) (table 2C). Subgroup C2 made up 24.2% and contained individuals almost all having diabetes (86.2%) and among whom smoking was common (41.4%). Finally, subgroup C3 (34.2%) consisted of smoking, non-diabetic men who were relatively young, with mean age of 55.5 (SD 3.4). This cluster solution had an average silhouette score of 0.5 corresponding to a ‘good’ cluster solution. In the bootstrap samples the BIC selected 2, 3, 4 or 5 clusters as the optimum number in 2.7%, 90.0%, 7.2%, and 0.1% of the samples, respectively. In 80.9% of the bootstrap samples no outliers were detected and <1% outliers were detected in the remaining 19.1% samples. The adjusted Rand index averaged over all bootstrap samples was 0.95 (SD 0.09).
